# Angiojet System Used in the Treatment of Submassive Pulmonary Embolism: A Case Report of Two Patients

**DOI:** 10.1155/2022/6867338

**Published:** 2022-08-23

**Authors:** Jinbo Liu, Tianrun Li, Wei Huang, Na Zhao, Hongwei Zhao, Hongyu Wang

**Affiliations:** ^1^Department of Vascular Medicine, Peking University Shougang Hospital, Beijing 100144, China; ^2^Vascular Health Research Center of Peking University Health Science Center, Beijing, China; ^3^Department of Interventional Vascular Surgery, Peking University Third Hospital, Beijing 100191, China

## Abstract

**Background:**

Massive or submassive pulmonary embolism (PE) carries a high mortality. Percutaneous mechanical thrombectomy using the Angiojet system is accepted for the treatment of PE. Here, we reported two submassive PE cases who were treated with the Angiojet system successfully, to provide some advice for the therapy of submassive PE.

**Method:**

Two patients with suffocation were admitted to our hospital. One patient was accompanied by lower blood pressure (20% lower than basal blood pressure) and higher pulmonary artery pressure (89 mmHg); the other patient had larger right ventricular transverse diameter (46 mm), decreased left ventricular end diastolic anteroposterior diameter (34 mm), and higher heartbeats (107 heartbeats per minute). Pulmonary artery computed tomography angiography showed bilateral pulmonary embolism.

**Result:**

The Angiojet system with a high-pressure jet spray pattern (urokinase 25 wiu + sodium chloride injection 50 ml) was used. Intravascular thrombolysis by urokinase (100 wiu/day for 1 day) was done after being back in the ward. And low molecular weight heparin was used in hospitalization, and rivaroxaban was used after discharge. Both patients were treated successfully. However, the level of platelet was significantly lower in one patient after Angiojet system usage and recovered to the preoperative level the next day. Another patient suffered from bradyarrhythmias during the usage of Angiojet, and bradyarrhythmias disappeared when the Angiojet system stopped. Pulmonary embolism was cured after 3 months in both patients.

**Conclusion:**

Angiojet could be a simple, safe, and well-tolerated treatment for massive or submassive PE. And hematocrit, platelet, kidney function, and heart rhythm should be monitored during perioperation.

## 1. Introduction

Pulmonary embolism (PE) especially massive or submassive PE carries a high mortality, associated with haemodynamic instability [1]. Thrombolytic therapy and surgical embolectomy are accepted for the treatment of massive PE [2]. However, these therapies might be accompanied by complications such as bleeding and surgical complications. So, percutaneous mechanical thrombectomy (PMT) using a variety of devices has been developed [3]. Akam-Venkata et al. [4] showed that catheter-directed therapy was an emerging alternative therapy for submassive and massive PE in children. And catheter-directed mechanical thrombectomy as the primary treatment for massive PE and submassive PE had a high rate of technical and clinical success in a high-risk patient population [5]. The Angiojet system is a device originally designed for thrombectomy in peripheral vessels or saphenous vein grafts [6]. And the Angiojet system showed an increasing use in emergency and election patients such as deep venous thrombosis [7]. A recent study showed that percutaneous rheolytic thrombectomy using the Angiojet system might be a valid treatment option for patients with massive or submassive pulmonary embolism with rapid and significant haemodynamic improvement and encouraging results at early and long-term follow-up [8].

Here, we reported two submassive PE cases who were treated with the Angiojet system successfully, to provide some advice for the therapy of submassive PE.

## 2. Case Presentation

**Figure 1:**
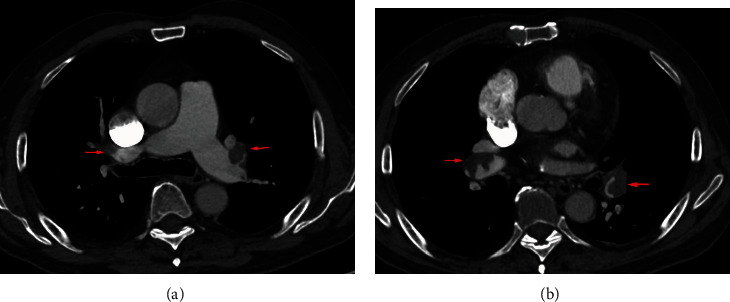
Computed tomography angiography (CTA) showed pulmonary embolism (yellow arrow) in the first patient (a, b).

**Figure 2:**
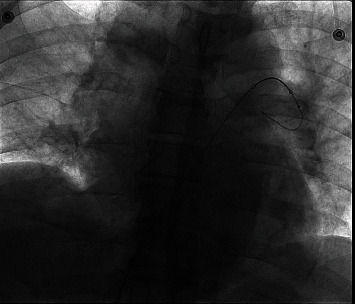
Angiographic imaging of Angiojet system in the first patient.

A patient (man, 87 years old) visited our hospital with suffocation for 3 days on August 31, 2019. This patient had a history of hypertension treated with valsartan amlodipine compound, with blood pressure 150/70 mmHg and 60 heartbeats per minute in peacetime. In addition, this patient had a history of lower extremity varicose veins without any therapy. We did some laboratory tests and examinations for this patient immediately after admission to the hospital. Whole blood count analysis revealed a white blood cell count of 9.1 × 10^9^/l (reference range 3.5-9.5 × 10^9^/l), haemoglobin level of 134 g/l (reference range 115-150 g/l), hematocrit level of 37.3% (reference range 40-50%), and a platelet count of 154 × 10^9^/l (reference range 125-350 × 10^9^/l). The results of NT-proBNP and creatine were 10671 pg/ml (reference range 0-1800 pg/ml) and 125.7 *μ*mol/l (reference range 20-98 *μ*mol/l), respectively. The level of troponin was normal. Further examination showed that autoantibodies were normal. The blood pressure was 120/80 mmHg, with 80 heartbeats per minute when admitted to the hospital. No thrombus was found in the lower extremity venous ultrasound examination. And pulmonary artery computed tomography angiography (CTA) showed bilateral pulmonary embolism (Figures 1(a) and 1(b)). Electrocardiogram showed right bundle branch block, and echocardiogram showed larger right ventricular transverse diameter (43 mm), decreased left ventricular end diastolic anteroposterior diameter (43 mm), and higher pulmonary artery pressure (89 mmHg). According to the clinical manifestations, blood pressure (20% lower than basal blood pressure), CTA, NT-proBNP, and echocardiogram, this patient was diagnosed with submassive pulmonary embolism. Pulmonary angiography was done on August 31, 2019, and the result confirmed pulmonary embolism. In addition, percutaneous mechanical pulmonary thrombectomy was done using the Angiojet system. The injection pattern by urokinase (urokinase 25 wiu + sodium chloride injection 50 ml) was used. And the main left pulmonary artery was treated with the Angiojet system for 20 s, and the main right pulmonary artery was treated with the Angiojet system for 15 s (Figure 2). However, this treatment was finished because of bradyarrhythmia, and bradyarrhythmia disappeared after catheter withdrawal. And we placed the catheter in the inferior vena cava; intravascular thrombolysis by urokinase (100 wiu/day, 1 day) was done after being back in the ward. And low molecular weight heparin was used in hospitalization, and rivaroxaban was used after discharge. In addition, whole blood count analysis revealed a white blood cell count of 11.5 × 10^9^/l (reference range 3.5-9.5 × 10^9^/l), haemoglobin level of 140 g/l (reference range 115-150 g/l), hematocrit level of 41% (reference range 40-50%), and a platelet count of 161 × 10^9^/l (reference range 125-350 × 10^9^/l) on September 1. The results of NT-proBNP and creatine were 801 pg/ml (reference range 0-1800 pg/ml) and 110 *μ*mol/l (reference range 20-98 *μ*mol/l) on September 9, respectively. And echocardiogram showed decreased right ventricular transverse diameter (42 mm), normal left ventricular end diastolic anteroposterior diameter (52 mm), and higher pulmonary artery pressure (72 mmHg) on September 11.

**Figure 3:**
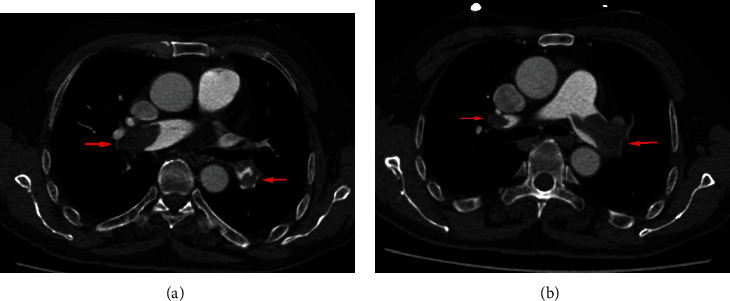
Computed tomography angiography (CTA) showed pulmonary embolism (yellow arrow) in the second patient (a, b).

**Figure 4:**
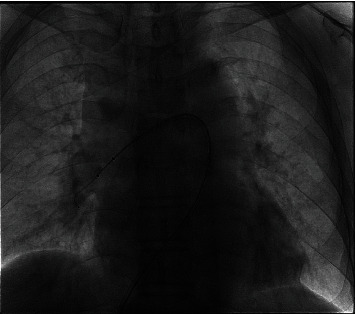
Angiographic imaging of Angiojet system in the second patient.

**Figure 5:**
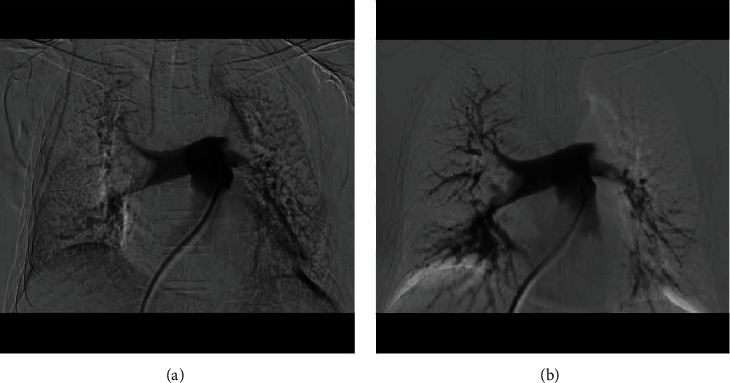
Angiographic imaging before (a) and after (b) Angiojet system therapy in the second patient.

Another patient (man, 67 years old) visited our hospital with chest tightness after activity for 1 day on September 10, 2019. This patient had a history of left lower limb trauma 2 months ago without history of hypertension, diabetes mellitus, and smoking. We did some laboratory tests and examinations for this patient immediately after admission to the hospital. Whole blood count analysis revealed a white blood cell count of 7.0 × 10^9^/l (reference range 3.5-9.5 × 10^9^/l), haemoglobin level of 138 g/l (reference range 115-150 g/l), hematocrit level of 39.1% (reference range 40-50%), and a platelet count of 103 × 10^9^/l (reference range 125-350 × 10^9^/l). The results of NT-proBNP and creatine were 5305 pg/ml (reference range 0-1800 pg/ml) and 76.1 *μ*mol/l (reference range 20-98 *μ*mol/l), respectively. The level of troponin was normal. Further examination showed that autoantibodies were normal. The blood pressure was 110/70 mmHg, with 107 heartbeats per minute when admitted to the hospital. An old thrombus was found in the left lower extremity venous ultrasound examination. And pulmonary artery computed tomography angiography (CTA) showed bilateral pulmonary embolism (Figures 3(a) and 3(b)). Electrocardiogram showed S_I_Q_III_T_III_ sign, and echocardiogram showed larger right ventricular transverse diameter (46 mm), decreased left ventricular end diastolic anteroposterior diameter (34 mm), and higher pulmonary artery pressure (44 mmHg). According to the clinical manifestations, CTA, NT-proBNP, and echocardiogram, this patient was diagnosed with submassive pulmonary embolism. Pulmonary angiography was done on September 10, 2019, and the result confirmed pulmonary embolism. In addition, percutaneous mechanical pulmonary thrombectomy was done using the Angiojet system (Figure 4). The injection pattern by urokinase (urokinase 25 wiu + sodium chloride injection 50 ml) was used. And the main left pulmonary artery was treated by the Angiojet system for 20 s, and the main right pulmonary artery was treated by the Angiojet system for 20 s. Thrombus aspiration was performed in both pulmonary arteries using a catheter by 50 ml syringe, and a massive thrombus was extracted (Figures 5(a) and 5(b)). And we placed the catheter in the inferior vena cava. Intravascular thrombolysis by urokinase (100 wiu/day, 1 day) was done after being back in the ward. And low molecular weight heparin was used in hospitalization, and rivaroxaban was used after discharge. In addition, whole blood count analysis revealed a white blood cell count of 6.6 × 10^9^/l (reference range 3.5-9.5 × 10^9^/l), haemoglobin level of 134 g/l (reference range 115-150 g/l), hematocrit level of 37.8% (reference range 40-50%), and a platelet count of 74 × 10^9^/l (reference range 125-350 × 10^9^/l) on September 11. And the level of platelet count was 125 × 10^9^/l (reference range 125-350 × 10^9^/l) on September 12. The results of NT-proBNP and creatine were 1391 pg/ml (reference range 0-1800 pg/ml) and 54.1 *μ*mol/l (reference range 20-98 *μ*mol/l) on September 12 and 22, respectively. And echocardiogram showed decreased right ventricular transverse diameter (29 mm), normal left ventricular end diastolic anteroposterior diameter (51 mm), and normal pulmonary artery pressure (26 mmHg) on September 26. The clinical characteristics of these two patients are shown in Table 1.

**Figure 6:**
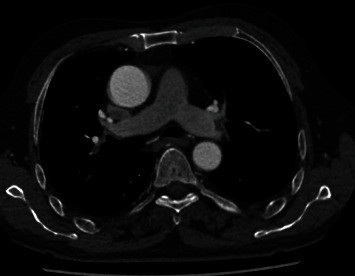
Computed tomography angiography (CTA) showed pulmonary embolism disappearance in the second patient.

In addition, pulmonary artery computed tomography angiography showed pulmonary embolism disappearance in the second patient after 3 months (Figure 6).

## 3. Intervention Process

The patient was supine, and the conventional bilateral inguinal region skin was disinfected by sterile drapes. 1% lidocaine anesthesia was done in the right femoral vein puncture point, the right femoral vein puncture, implanted with a 5 f sheath pipe. By going through the sheath pipe, the pigtail catheter was placed at the level of L5 by an ultrasmooth guide wire. The patency of inferior vena cava and bilateral renal vein open position were confirmed by angiography. The tail catheter and sheath tube were removed, and the ultrasmooth guide wire was retained. The special long sheath for the filter was replaced using an ultrasmooth guide wire, and the inferior vena cava filter (DENALI) was implanted in the lower segment of the renal vein and the vertical segment of the inferior vena cava. The long sheath of the filter was replaced by a soft sheath tube with a hard guide wire, and the sheath tube was placed near the heart position of the inferior vena cava. Guided by the sheath tube and ultrasmooth guide wire, the pigtail catheter was placed in the main pulmonary artery, and pulmonary angiography was performed to confirm pulmonary embolism. The stiff guide wire through the pigtail catheter at the main left pulmonary artery was placed, the pigtail catheter was removed, and the sheath catheter was placed through the guide wire at the main pulmonary artery. The Angiojet system was placed in the main pulmonary artery through the sheath tube, and the injection device was used for thrombolysis treatment. And then, the Angiojet system was removed, and the catheter was placed into the main pulmonary artery through a sheath tube, with a 50 ml syringe at the end of the catheter for thrombus aspiration. Finally, the pigtail catheter was placed through the sheath at the main pulmonary artery for reexamination. The sheath was withdrawn to the inferior vena cava L5 level under the support from the thread. And we placed the catheter in the inferior vena cava. Intravascular thrombolysis by urokinase (100 wiu/day, 1 day) was done after being back in the ward. After 24 hours, the catheter and sheath tube were pulled out, and local pressure was applied for 10 minutes. Then, the bandages were bandaged. The bandage was removed, and the patient could move out of bed after 24 hours.

## 4. Discussion

Pulmonary embolism (PE) especially massive or submassive PE associated with haemodynamic instability was a threatening factor for human life. Early aggressive treatment restoring patency of occluded pulmonary arteries was the principal factor affecting mortality in massive PE [9]. According to the guidelines of the European Society of Cardiology, shock or systemic hypotension is an accepted indication for urgent thrombolysis in patients with massive PE, and surgical or endovascular thrombectomy can be an option in selected cases [10].

The Angiojet system is a device originally designed for thrombectomy in peripheral vessels or saphenous vein grafts. The Angiojet system incorporates mechanical fragmentation, pharmacologic lysis, and rheolytic aspiration of clots. And many studies confirmed the successful usage of the Angiojet system in deep venous thrombosis [11].

However, there was little research about the usage of the Angiojet system in PE, and most research was case reports of small samples, and this might be caused by the characteristics of massive or submassive PE. A previous study showed that Angiojet system technical success was obtained in 92.2% of PE patients, with a significant improvement in obstruction, perfusion, and Miller indexes [12]. The Angiojet system procedure might be safely performed in PE patients with cardiogenic shock [13]. Another study about the combined therapy including the catheter-directed thrombolysis method using adjunctive power-pulse spray technique using the Angiojet system showed that there were no major or minor adverse events and no procedure-related complications in treating massive and submassive acute PE. And this study offered several potential advantages compared with current options, allowing safer and faster thrombus resolution [14]. Guo et al. showed that the Angiojet system combined with catheter fragmentation was successfully used in the treatment of massive PE [15]. There were some studies about massive PE patients with thrombolytic therapy contraindicated. Zuin et al. reported a patient with acute PE, heparin-induced thrombocytopenia type II, and recent ischemic stroke, and this patient was successfully treated with rheolytic thrombectomy by the Angiojet system, suggesting the usefulness of the Angiojet system in treating acute PE in clinically difficult scenarios, especially when thrombolytic therapy is contraindicated [16]. Vecchio et al. reported a patient who developed acute intermediate-risk PE, with right ventricular dysfunction and major myocardial necrosis, and this patient was successfully treated with the Angiojet system [17]. These studies confirmed at early and long-term follow-up the effectiveness and safety of rheolytic thrombectomy using the Angiojet system for PE [18, 19].

However, there might be some complications with the usage of the Angiojet system. First, Angiojet will cause not only clot dissipation, with its high-pressure jet spray, but also some destruction of blood cells, so hematuria was a corollary and “self-limited” side effect on patients treated with Angiojet [20]. Second, a recent study showed that procedure-related anemia was one significant minor complication [21]. Third, another study showed that bradyarrhythmia might appear during the usage of the Angiojet system. This might be related to the effects of the hydrodynamic jets on stretch-activated receptors present on the vascular endothelium, and pretreatment with gadolinium or streptomycin might prevent activation of these receptors [22]. Fourth, Angiojet usage was an independent risk factor for acute kidney injury, and this might be related to haemolysis from the device [23]. And a recent study found that Angiojet system usage would raise the risk of postoperative acute kidney injury, especially in patients with a history of major surgery within 3 months of endovascular intervention, and hematocrit drop > 14% might indicate upcoming acute kidney injury [24]. In addition, pulmonary artery CTA and interventional therapy required the participation of contrast material, which might cause renal insufficiency, especially in the elderly. And troponin values would be expected to increase in a patient with impaired renal function. However, we did not measure postoperative troponin levels in the present study, and this process should be perfected in future cases. In the present study, our results showed that there was no difference in the level of hematocrit, haemoglobin, and kidney function before and after Angiojet system usage. However, the level of platelet was significantly lower in one patient after Angiojet system usage, and the level of platelet recovered to the preoperative level the next day. We thought this might be related to high-pressure jet spray and mechanical damage of the Angiojet device, and this complication was first reported so far. In addition, one patient suffered from bradyarrhythmias during the usage of Angiojet, and bradyarrhythmias disappeared when the Angiojet system stopped, similar to a previous study.

In conclusion, Angiojet could be a simple, safe, and well-tolerated treatment for massive or submassive PE. And hematocrit, platelet, kidney function, and heart rhythm should be monitored during perioperation.

## Figures and Tables

**Table 1:** Clinical characteristics in these two patients.

Characteristics	Patient 1		Patient 2	
	Pre-Angiojet therapy	Post-Angiojet therapy	Pre-Angiojet therapy	Post-Angiojet therapy
White blood cell (×10^9^/l)	9.1	11.5	7	6.6
Haemoglobin (g/l)	134	140	138	134
Hematocrit (%)	37.3	41.1	39.1	37.8
Platelet (10^9^/l)	154	161	103	74
NT-proBNP (pg/ml)	10671	801	5305	1391
Creatine (*μ*mol/l)	125.7	110	76.1	54.1
Blood pressure (mmHg)	120/80	140/80	110/70	126/70
Heart rate (per minute)	80	65	107	74
Right ventricular transverse diameter (mm)	43	42	46	29
Left ventricular end diastolic anteroposterior diameter (mm)	43	52	34	51
Pulmonary artery pressure (mmHg)	89	72	44	26

## Abbreviations

PE: Pulmonary embolism
PMT: Percutaneous mechanical thrombectomy.
